# Identification of Education Models to Improve Health Outcomes in Arab Women with Pre-Diabetes

**DOI:** 10.3390/nu11051113

**Published:** 2019-05-18

**Authors:** Rasha Al-Hamdan, Amanda Avery, Andrew Salter, Dara Al-Disi, Nasser M. Al-Daghri, Fiona McCullough

**Affiliations:** 1 Division of Nutritional Sciences, School of Biosciences, University of Nottingham, Nottingham NG7 2RD, UK; amanda.avery@nottingham.ac.uk (A.A.); andrew.salter@nottingham.ac.uk (A.S.); fiona.mccullough@nottingham.ac.uk (F.M.); 2 Department of Community Health Sciences, College of Applied Medical Sciences, King Saud University, Riyadh 11451, Saudi Arabia; daldisi@ksu.edu.sa; 3 Chair for Biomarkers of Chronic Diseases, Biochemistry Department, College of Science, King Saud University, Riyadh 11451, Saudi Arabia; ndaghri@ksu.edu.sa

**Keywords:** pre-diabetes, lifestyle, Arabs, intervention

## Abstract

Few evaluations of interventions to delay or prevent type 2 diabetes mellitus (T2DM) in Saudi Arabia (SA) have been undertaken. The present study evaluates the impact of a 6-month intensive lifestyle modification intervention delivered in primary care. Females from SA with prediabetes, aged 18–55 years, were recruited with 190 participants eligible following screening and randomly allocated to receive a 3-month one-on-one, intensive lifestyle modification (intervention group (IG) *n* = 95) or standard guidance (control group (CG) *n* = 95). Participants completed questionnaires including demographic, dietary and physical activity data. Blood samples were collected at baseline, 3 and 6 months. A total of 123 (74 IG (age 40.6 ± 9.8 years; body mass index (BMI) 31.2 ± 7.0 kg/m^2^) and 49 CG (age 40.6 ± 12.7 years; BMI 32.3 ± 5.4 kg/m^2^)) participants completed the study. After 6 months, haemoglobin A1c (HbA1c; primary endpoint) significantly improved in the IG than CG completers in between-group comparisons (*p* < 0.001). Comparison between groups showed significant improvements in overall energy intake, total and high density lipoprotein (HDL)-cholesterol in favour of IG (*p*-values < 0.001, 0.04 and <0.001, respectively). BMI and weight change were not clinically significant in between group comparisons. A 6-month, intense one-on-one intervention in lifestyle modification significantly improves glycaemic and cardio metabolic profile of females living in SA with pre-diabetes delivered in a primary care setting. Longer duration studies, using the same intervention, may determine whether a meaningful weight loss secondary to improved diet can be achieved.

## 1. Introduction

Diabetes mellitus (DM) is a chronic disease characterised by the body’s inability to produce insulin or use it efficiently [1]. The global prevalence of DM in the adult population was 8.4% or 451 million as of 2017, and is expected to reach as much as 10% or 693 million in 2045 [2]. Although different treatments for DM are available, prevention remains key in slowing down the escalating prevalence of the disease. The target population for DM prevention are those with prediabetes, defined as having an impaired fasting glucose (IFG) and/or impaired glucose tolerance (IGT) and/or HbA1c 5.7%–6.4% [3]. A recent review by Yip and colleagues indicated that the prevalence of prediabetes using the combined IFG/IGT are elevated, but not statistically different in both Caucasian and Asian ethnicities (19.8% vs. 20.5%, respectively) [4].

Saudi Arabia (SA) is not immune to DM and other chronic non-communicable diseases. Type 2 diabetes mellitus (T2DM) in particular, is an important public health threat in SA with prevalence rates increasing over time and being increasingly more common among urban women (33%) and older adolescents (10.8%) [5]. There is evidence to suggest that individuals from SA with DM have poor knowledge about the disease [6], its risk factors and preventive measures [7], symptoms [8] and resulting macro- and micro-vascular complications [9]. Aside from the conventional T2DM risk factors such as age, obesity and sedentary lifestyle, the population in SA appears to have a genetic predisposition to T2DM, given its high prevalence of consanguineous marriages and gestational diabetes mellitus (GDM) [10,11]. It is estimated that the economic burden of T2DM in SA in 2014 was SR17 billion and higher at SR27 billion if the undiagnosed pool are included and SR43 billion if the prediabetes population is left untreated [12]. Given these costs, it makes sense that effective diabetes management programs, whether prevention or management, should be successfully implemented in SA.

Landmark studies on diabetes prevention such as the Diabetes Prevention Program (DPP) in the USA, the Finnish Diabetes Prevention Study in Finland and the Da Qing Impaired Glucose Tolerance and Diabetes Study in China all used lifestyle modification strategies aimed at reducing weight through physical activity and improving the diet decreased intake of fat and increased intake of fibre [13,14,15]. More recently, compelling evidence in significantly improving HbA1c in the pre-diabetic population was also observed using individualised medical nutrition therapy [16]. There is available information, although limited, on T2DM prevention studies undertaken in SA. In a 6-week T2DM education program carried out at the primary health clinics in Dammam, women at risk of or diagnosed with T2DM (*n* = 35 including dropouts) were assigned to one of two groups; an intervention group that participated in an education program tailored to their cultural and religious contexts; and a control group that received the usual standard care for people with diabetes in SA. Compared with the control group, the intervention group had a significant improvement in health-related quality of life and blood glucose levels. The intervention group also became more disciplined in monitoring their blood glucose [17]. Further studies done locally showed that successful management of DM relies with the improvement of the patient’s level of knowledge about diabetes, increasing the level of compliance by as much as 78% [18,19]. An evaluation found that patients in SA were more compliant to dietary instructions than to directions on exercise [20]. Other studies have shown that more than one-third and nearly half of the participants did not adhere to diet and exercise recommendations, respectively, with non-adherence to exercise as more common than non-adherence to diet [18,21].

Educational programs in general seem to have a clinically significant beneficial effect among the T2DM population in terms of improved glycaemic control. These programs include intensified self-monitoring of blood glucose with education [22], tailored interventions specific for special holidays such as Ramadan [23], cultural contexts [17] and the prevention of diabetic complications such as diabetic foot [24]. Pre-Ramadan educational programs have been found to have less desirable outcomes in terms of attendance [25].

There is limited evidence from clinical trials that lifestyle modification through diet and physical activity reduces and even prevents the onset of diabetes among high risk ethnic groups [26]. To date, no intensive lifestyle program for women in SA at risk of developing T2DM has been conducted in primary care. This is important since women in SA have unique barriers not present in other cultures that can predispose them to sedentary lifestyle (e.g., compulsory wearing of abaya or full length outer garment in public, gender segregation and activities that are mostly indoors). Furthermore, Saudi women are disproportionately much more sedentary than men due to restrictions in outdoor activities [27]. Accordingly, an Arab Muslim woman is twice more likely to be physically inactive than a non-Arab, non-Muslim woman, increasing their risk for pre-diabetes and ultimately T2DM [28]. The present study therefore aims to explore for the first time, the impact of a 6-month personalised lifestyle intervention provided one-to-one to Saudi women with pre-diabetes as compared to standard care in a primary care setting.

## 2. Materials and Methods

### 2.1. Study Population and Sampling Method

In this interventional study, females living in SA aged 18–55 years old who were either overweight (body mass index (BMI) 25–29.9 kg/m^2^) or obese (BMI ≥ 30 kg/m^2^), attending the primary health care centres (PHCCs) in Riyadh, SA were randomly selected and assessed for the presence of prediabetes. A participant was considered pre-diabetic if HbA1c level was ≥5.7% to <6.5%, as recommended by the American Diabetes Association (ADA) guidelines [29]. Pregnant women, not of Saudi origin, those with T1DM or T2DM, and those with chronic medical co-morbidities, such as cancer, liver, kidney and thyroid diseases as well as malignant cases were excluded.

The study was carried out from December 2016 to June 2017, and commenced after receiving an approval from the Ethics Committee of the College of Science, KSU (Ethic No. 8/25/220355, approved last May 13, 2011) in accordance with the protocol and the ethical principles that have their origin in the declaration of Helsinki, as well as with the guidance on good clinical practice. In order to facilitate the implementation of the study in Saudi Arabia, the team was supported by the Department of Nutritional Science in University of Nottingham and the Chair for Biomarkers of Osteoporosis, KSU in Riyadh, SA. A consent form was signed by patients who were to participate in the study. Confidentiality was guaranteed.

### 2.2. Randomisation

Following screening, 190 participants were randomly assigned using the Excel RAND function. Selected subjects were then contacted and invited to participate in the study, then randomly assigned to receive either intensive lifestyle intervention group (IG, *n* = 95) or to the control group (CG, *n* = 95). 

### 2.3. Data Collection

Baseline assessments included anthropometric measurements, blood pressure, blood sample tests and submission of self-administered questionnaires which included demographics, medical history and physical activity, taking into consideration the cultural context (in-door physical activities and non-gender mixing). Assessment of dietary intake was done using a food frequency questionnaire (FFQ) which consisted of 140 food items developed to capture the dietary habits of Saudis. This FFQ had been tested for internal consistency, test-retest reliability, completeness of food list, and criterion validity [30]. The assessments were repeated at 3-month intervals (at baseline, 3 and 6 months) in both groups. A total of 123 completed questionnaires were collected in closed envelopes from all participating PHCCs and sent to University of Nottingham for data analysis. 

Anthropometric measurements were conducted by medical staff trained by the principal investigator. Height and weight were measured with light clothing without shoes. Waist circumference was measured at the umbilical level without clothes after exhaling in a relaxed standing position. BMI was calculated by dividing weight in kg over height in meters squared.

Blood pressure was measured at each visit after sufficient rest. Fasting blood samples were collected using a sterile vacutainer blood collection apparatus. Variables included the measurement of haemoglobin A1c (HbA1c) in whole blood, as well as fasting glucose and lipid profiles in serum (total and high-density lipoprotein cholesterol and triglyceride levels). Glycaemic and lipid profile were measured by auto-analysers at the in-house laboratory of each local study centre. 

Outcomes include: HbA1c levels (primary outcome), weight change and lipid profile. The questionnaires were administered by a certified dietitian, physician, nurse, physical therapist and diabetes educator along with a coordinator for arranging patients’ appointments. A local language (Arabic) was used as the main language in the study. A total of 123 completed questionnaires (out of 190) questionnaires distributed were collected from 74 participants in the intervention group and 49 participants in the control group. 

### 2.4. Intervention

The 3-month education program was launched in a primary health clinic close to where most participants lived. A small pilot sized study was carried out with five females from SA with prediabetes in order to assess the tools provided were understood by participants and ensured that questionnaires were adapted to the cultural contexts of women in SA.

**Figure 1:**
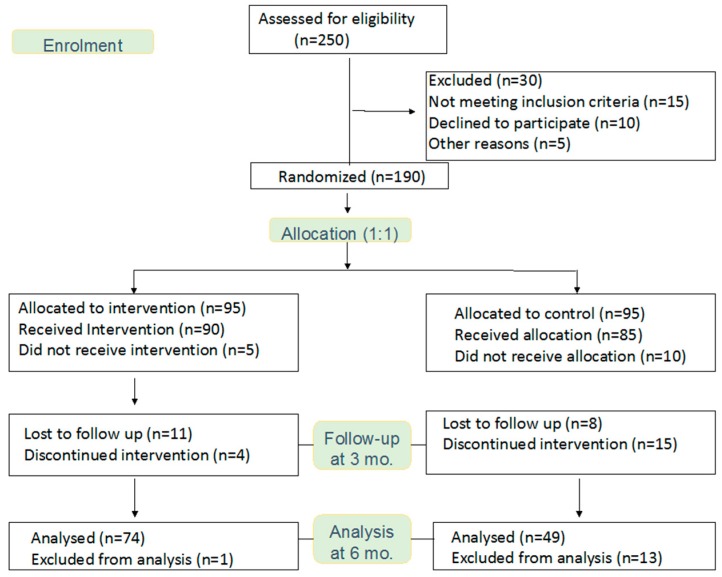
Flow chart.

All groups were given a session on medical nutrition therapy by a registered dietitian. Participants in the IG received dietary counselling and were advised individually through personal communication to reduce weight (at least 5% from baseline weight), increase physical activity (4 h/week), and consume a moderate fat (total fat 30% of total energy consumed and saturated fat 10% of total energy consumed), high fibre (15 g/1000 kcal) diet. In addition, the IG was given individualised dietary counselling by the study nutritionist and lifestyle education sessions every two weeks for 3 months to reinforce lifestyle changes. Physical activities were also encouraged and monitored every two weeks for 3 months in the IG. In the CG, lifestyle intervention instructions were given as a printed booklet. Furthermore, dietary intake and physical activity were noted only at baseline and every 3 months. The CG did not receive lifestyle education sessions, dietary counselling and on demand support system. Both groups were followed-up 3 months after completion of the education program. Figure 1 shows the flow chart of the participants during the study period. Table 1 shows the difference in interventions given in groups.

### 2.5. Data Analysis

A G-power test was done to calculate the estimated sample size needed. Given α = 5% and a standard deviation of 0.21 (anticipated change in HbA1c), a sample size of *n* = 49 per arm has 80% power to detect a moderately strong association between dietary habits, physical activity, anthropometric, biochemical measurements and health education (at a confidence level of 95%). 

Data were analysed using SPSS version 22.0 (SPSS Inc., Chicago, IL, USA). Confidence intervals (95% CIs) were used to examine the pre/post differences of the sample means of physical measurements and biochemical parameters, between the two groups. Frequencies were presented as *N* (%) and continuous variables were expressed as mean ± standard deviation. Differences between categorical variables were determined using Chi-Square tests. Differences between IG and CG were determined using Independent Student’s *T*-test. Repeated measures analysis of variance (repeated measures ANOVA) was used to determine differences across time points and in within group comparisons using per protocol analysis. Mixed method analysis of covariance (ANCOVA) was used to determine between group differences with adjustments for significant baseline covariates differences. All non-normal variables were transformed prior to parametric testing. A Levene’s test verified the equality of variances (*p* > 0.05). A *p*-value <0.05 was considered statistically significant. 

## 3. Results

### 3.1. Study Participants 

A total of 250 participants from five primary health care centres in Riyadh, SA were assessed for eligibility. A total of 190 participants met the inclusion criteria and were randomly allocated; 95 participants in the IG, and 95 participants allocated in the CG. After 6 months, data was available for 74 participants completing the IG and 49 participants completing the CG. Participation rate was 58.5% for the IG and 41.5% for the CG. Figure 1 illustrates the recruitment process and attrition at different stages. 

### 3.2. General Characteristics

More than half of the study participants in both groups were living in the Eastern, Western, and Middle province of SA, but with slightly more of IG living in urban areas. All participants are females with pre-diabetes (HbA1c ≥5.7% to <6.5%) aged 18 and 55 years. A total of 123 completed questionnaires were collected from participants who completed the intervention or control arm and for whom 6-month data was available. The mean age of all 123 subjects was 40.6 ± 9.8 years. The majority of the participants who participated were married (69.9%), mostly to a first degree relative (54.5%). One out of four participants said that they had never been pregnant (25.2%). Around 31% had a history of GDM (37% in IG; 0 in CG). The rest of the characteristics are found in Supplementary Table S1.

### 3.3. Baseline Clinical and Dietary Intake Differences between Groups

Table 2 shows the baseline characteristics of IG and CG. No significant differences were found in all anthropometric measures as well as glycaemic parameters. In the lipid profile however, CG had a significantly lower total cholesterol and high density lipoprotein (HDL)-cholesterol than IG (*p*-values 0.04 and <0.001, respectively). Dietary intake in the IG was significantly higher than the CG with regards to fat energy (%) (*p* = 0.02). The dietary intake of the CG on the other hand, was significantly higher than the IG in terms of total fat (DRI) (*p* = 0.001) and protein in grams. No significant differences were observed for the rest of the macro- and micronutrients, including energy intake (Table 2).

### 3.4. Clinical Differences between Groups Over Time

Table 3 shows the within and between group comparisons over time. In the IG, there was a significant but modest decrease in BMI after 6 months of intervention compared to baseline and after 3 months (*p* < 0.05). Percentage weight change was also noted to be significant after 6 months (*p* < 0.05). In parallel, significant reductions were also observed in waist circumference over time with 6 months having significantly reduced as compared to baseline and after 3 months (*p*-values < 0.05). Hip circumference was significantly lower after 6 months than baseline (*p* < 0.05). No significant changes were observed in waist-hip ratio, systolic and diastolic blood pressure. In CG, no significant changes were observed in all anthropometric measures. Between group comparisons indicated a clinically significant improvement in waist-hip ratio (*p* = 0.04) and systolic blood pressure (*p* = 0.01) in favour of the IG.

With regards to the glycaemic profile, within group comparison showed a significant reduction in both fasting glucose and HbA1c in IG over time, with 6 months being significantly lower than baseline and 3 months (*p*-values <0.05). Serum fasting glucose did not change over time in CG and a significant increase was observed in HbA1c, with 6 months being significantly worse than baseline and 3 months (*p* < 0.05). Between group comparisons showed a clinically significant difference in HbA1c levels in favour of IG (*p* < 0.001).

In the lipid profile, no significant difference was observed in circulating triglycerides over time in IG. However, a significant reduction in both total and LDL-cholesterol were observed, with 6 months being significantly lower than baseline and 3 months, as well as 3 months being significantly lower than baseline (*p*-values < 0.05). Furthermore, there is a significant increase in HDL-cholesterol, with 3 months being significantly higher than baseline and 6 months being significantly higher than baseline and 3 months (*p*-values < 0.05). In CG, modest but significant improvements were also noted in triglycerides and total cholesterol, with 6 months being significantly lower than baseline (*p*-values < 0.05). Between group comparisons showed a clinically significant decrease in total cholesterol (*p* = 0.04) and a clinically significant increase in HDL-cholesterol (*p* < 0.001) in favour of IG. Lastly, no significant differences were noted in physical activities of both the IG and CG in within and between-group comparisons.

With regards to the diet, energy intake was significantly reduced in IG (*p* < 0.05), reflected by decreased carbohydrate (g), protein (g) and fat (g) intake in IG after 3 months and 6 months (*p*-values < 0.05). Within group comparisons in CG showed a significant decrease in carbohydrate (% energy) and fat (grams and % energy) intake over time, particularly after 6 months as compared to baseline (*p*-values < 0.05). Energy intake (actual kilocalories) over time increased modestly after 6 months compared to baseline (*p*-values < 0.05).

As shown in Table 3, between-group comparisons, after adjusting for baseline covariates, showed a clinically significant decrease in carbohydrate (g) intake in favour of IG and a clinically significant increase in protein (grams and % energy) consumption in favour of CG. Fat energy (%) significantly decreased in CG than IG (*p* = 0.002). Energy intake (actual kilocalories) over time was significantly lower in IG than CG (*p*-values < 0.001).

## 4. Discussion

The main finding of the present study is that a 6-month, personalised, intensive, one-on-one approach in the lifestyle modifications of Saudi women with prediabetes can lead to significant improvements in the cardio metabolic profile, primarily through improvement in glycaemic indices, as compared to standard care. These effects were seen in the IG and not in the CG. This type of intervention, considered first in SA, highlights the importance of augmented personal contact to high-risk populations, including people with prediabetes, to increase the chances of success in any lifestyle modification program implemented in this population. It is also worth mentioning that these programs were conducted in primary care centres because these are the frontline caretakers in providing medical services in SA. The present findings are in alignment with a similar interventional study done in Saudi patients with and without components of the metabolic syndrome [31]. In a recent meta-analysis review by Pillay and colleagues, self-management education programs in diabetes were observed to be of little benefit if contact of delivery personnel to patients were less than 10 h [32]. This has been the case with the CG.

In the present study, both groups were unable to improve their activity levels. Whilst the IG was not able to achieve a greater weight loss, they did improve their diet quality. This improved diet quality may improve weight management longer term, but not seen at six months. Likewise, increasing physical activity given only as advice without actual monitoring may not be the best strategy in Arab women with prediabetes. Many intervention studies and randomised controlled trials (RCTs) confirm that manipulation of the diet alone among individuals with pre-diabetes significantly reduces risk of DM [33,34]. It has been recommended by the Academy of Nutrition and Dietetics that individualised medical nutrition therapy (MNT), the same dietary intervention used in the study, be implemented for individuals with prediabetes [35].

With regards to the diet, there was an overall decrease in energy intake, dietary carbohydrate, protein and fat in the IG post-intervention. These significant decreases in macronutrient intake was not observed in CG and in fact, the opposite trend was observed. The effects of low-calorie diet in weight reduction and overall improvement in insulin sensitivity is well documented [36,37]. In a recent review, dietary restriction has also been proven to improve cardio metabolic profile and is most beneficial among non-diabetic individuals with other risk factors during the holy month of Ramadan [38]. The present findings are in alignment with a similar intervention study done by Coppell and colleagues in the primary care centres of New Zealand where a structured dietary intervention that included dietary assessment, goal setting, dietary advise sessions and standard advice for physical activity for 6 months led to a significant decrease in the glycaemic profile of pre-diabetic participants and the opposite was observed in the CG [39]. The difference however in the present study is that the latter had a clinically significant difference in between group comparisons which was not observed in the previous study.

The clinically significant improvement in HbA1c, the primary outcome of the present study, in favour of IG, confirms that modifying dietary behaviour alone, even in the absence of physical activity, can have a meaningful impact in improving HbA1c levels [40]. A recent meta-analysis has also demonstrated for the first time that the greater the carbohydrate restriction, the greater glucose lowering can be achieved, at least in the short term and independent of weight change [41].

The present study is one of the first in SA to successfully conduct an intensive lifestyle program for Arab women at risk of developing T2DM at the primary care level using HbA1c as its primary endpoint. Primary care centres have always been the frontline of defence when it comes to everyday diseases within a given community. It makes sense therefore that the overall community health is tied significantly to how efficient its primary care centre functions. The overall care and management of T2DM patients in SA at the primary care level, while in progress and improving, still has plenty of room for improvement from prevention to treatment. The referral system alone from primary to secondary hospitals for T2DM patients was deemed “unsafe” [42] and screening for the most common complications such as diabetic retinopathy in primary care centres was found unsatisfactory [10]. Furthermore, it has been recently highlighted that both community diabetic centres and primary care centres in the country failed to improve both HbA1c and BMI over a 5-year period [43]. It is clear that much needs to be done in primary care centres in SA, if the overall picture of diabetes management is to improve in the country. The results of the present study among pre-diabetics confirm that in the presence of a more vigilant and pro-active primary care team and focused care, preventive healthcare is very much possible in these settings.

## 5. Conclusions

In summary, a 6-month primary-care led one-on-one advice on intensive lifestyle modification program focusing on dietary management, even with limited physical activity, is effective in improving glycaemic and cardio metabolic profiles of pre-diabetic adult females living in SA. However, no benefits were seen in weight loss compared to CG. Further work exploring a structured physical activity programme alongside dietary education is required. Collectively, the present study provided novel information about the suitable lifestyle intervention among the pre-diabetic population in SA and will have important implications for T2DM preventive policies and recommendations.

## Figures and Tables

**Table 1:** Interventions given to groups.

Lifestyle Intervention	IG	CG
Baseline Reduce weight (5% from baseline weight) Exercise (4 h/week) Reduce fat intake (30% of total energy and saturated fat 10% of total energy) Increase fibre intake (15 g/1000 kcal) diet	Explained by registered dietitian individually	Given as a booklet to the group
Bimonthly lifestyle education sessions for 3 months	✔	None
Dietary counselling	Every two weeks for 3 months	None
Dietary intake record		Baseline and after 3 months
Physical activity record		
Mode of follow-up	Individualised	Group
On demand support system	✔	None
Blood extraction	Baseline and every 3 months	
Anthropometrics		

IG, intervention group; CG, control group.

**Table 2:** Baseline anthropometric, clinical, and dietary intake comparison according to groups.

Parameter	IG	CG	*p*-Value
Anthropometrics			
BMI (kg/m^2^)	31.2 ± 7.0	32.3 ± 5.4	0.38
Waist (cm)	88.6 ± 13.5	91.3 ± 14.8	0.29
Hips (cm)	105.2 ± 13.4	105.4 ± 14.6	0.96
Waist-Hip Ratio	0.84 ± 0.08	0.87 ± 0.08	0.09
Systolic BP (mmHg)	122.6 ± 11.4	127.9 ± 16.7	0.06
Diastolic BP (mmHg)	77.8 ± 10.8	75.6 ± 11.0	0.29
Glycaemic Profile			
Glucose (mmol/L)	5.6 ± 0.9	5.4 ± 2.3	0.54
HbA1c (%)	6.0± 0.3	6.1 ± 0.29	0.052
Lipid Profile			
Triglycerides (mmol/L)	1.4 ± 0.7	1.6 ± 1.0	0.21
Total Chol (mmol/L)	5.1 ± 1.0	4.8 ± 0.8	0.04
HDL-Chol (mmol/L)	1.3 ± 0.3	1.0 ± 0.4	<0.001
LDL-Chol (mmol/L)	3.2 ± 0.9	3.0 ± 0.8	0.18
Energy Intake			
Energy (kilocalories)	2490.1 ± 522.9	2593.5 ± 519.8	0.28
Macronutrients			
Carbohydrate (g)	389.9 ± 94.8	415.2 ± 80.0	0.12
Carbohydrate Energy (%)	62.8 ± 10.3	64.5 ± 7.5	0.31
Protein (g)	95.9 ± 58.2	109.4 ± 44.8	0.17
Protein Energy (%)	15.3 ± 6.7	16.8 ± 5.5	0.18
Fat (g)	60.8 ± 26.8	55.0 ± 26.0	0.23
Fat Energy (%)	21.9 ± 8.5	18.6 ± 6.2	0.02

Data presented as mean ± SD; significant at *p* < 0.05. BMI, body mass index; BP, blood pressure; Chol, cholesterol; DRI, Dietary Reference Intake.

**Table 3:** Within and between group comparisons according to anthropometric, clinical, dietary intake and lifestyle across time points in all completing participants.

Parameter	Intervention			Control			*p*-Value
	Baseline	3 Months	6 Months	Baseline	3 Months	6 Months	
Anthropometrics							
BMI (kg/m^2^)	31.2 ± 7.0	31.1 ± 7.0	30.4 ± 7.1 ^ab^	32.3 ± 5.4	32.0 ± 5.3	31.5 ± 5.0	0.38
% Weight change	-	−0.14 (6.3)	−2.28 (10.7) ^b^	-	−0.62 (5.2)	−1.82 (10.0)	0.99
Waist (cm)	88.6 ± 13.5	87.5 ± 14.0	85.9 ± 14.0 ^ab^	91.3 ± 14.8	92.6 ± 13.8	91.4 ± 15.0	0.09
Hips (cm)	105.2 ± 13.4	104.3 ± 13.0	103.3 ± 13.6 ^a^	105.4 ± 14.6	106.5 ± 13.0	105.9 ± 14.4	0.51
Waist-hip ratio	0.84 ± 0.08	0.84 ± 0.08	0.83 ± 0.09	0.87 ± 0.08	0.87 ± 0.06	0.86 ± 0.08	0.04
Systolic BP (mmHg)	122.6 ± 11.4	122.5 ± 8.7	121.9 ± 9.3	127.9 ± 16.7	127.6 ± 17.4	127.4 ± 13.6	0.01
Diastolic BP (mmHg)	77.8 ± 10.8	77.6 ± 7.5	78.3 ± 10.7	75.6 ± 11.0	77.8 ± 11.1	80.1 ± 8.3	0.96
Glycaemic Profile							
Glucose (mmol/L)	5.6 ± 0.9	5.6 ± 0.8	5.2 ± 0.8 ^ab^	5.4 ± 2.3	5.3 ± 1.3	5.4 ± 1.1	0.79
Hba1c (%)	6.0 ± 0.3	6.0 ± 0.3	5.8 ± 0.3 ^ab^	6.1 ± 0.29	6.1 ± 0.4	6.3 ± 0.4 ^ab^	<0.001
Lipid Profile							
Triglycerides (mmol/L)	1.4 ± 0.7	1.4 ± 0.7	1.4 ±0.6	1.6 ± 1.0	1.5 ± 0.6	1.4 ± 0.5 ^a^	0.31
Total Chol (mmol/L)	5.1 ± 1.0	5.0 ± 1.0	4.7 ± 1.0 ^ab^	4.8 ± 0.8	4.6 ± 0.8	4.5 ± 0.8 ^a^	0.04
HDL-Chol (mmol/L)	1.3 ± 0.3	1.4 ± 0.4 ^a^	1.8 ± 0.5 ^ab^	1.0 ± 0.4	1.0 ± 0.3	1.1 ± 0.3	<0.001
LDL-Chol (mmol/L)	3.2 ± 0.9	3.0 ± 0.9 ^a^	2.3 ± 1.1 ^ab^	3.0 ± 0.8	2.9 ± 0.7	2.8 ± 0.8	0.73
Energy Intake							
Energy kilocalories	2490 ± 523	2226 ± 488 ^a^	2055 ± 462 ^ab^	2594 ± 520	2724 ± 581 ^a^	2708 ± 536 ^a^	<0.001
Macronutrients							
Carbohydrate (g)	389.9 ± 94.8	347.9 ± 93.4 ^a^	322.2 ± 93.7 ^ab^	415.2 ± 80.0	415.8 ± 85.4	416.3 ± 81.6	<0.001
CHO energy (%)	62.8 ± 10.3	62.4 ± 10.2	62.3 ± 9.9	64.5 ± 7.5	61.7 ± 8.5 ^a^	62.2 ± 10.2 ^a^	0.86
Protein (g)	95.9 ± 58.2	87.5 ± 52.0 ^a^	80.2 ± 42.3 ^ab^	109.4 ± 44.8	143.2 ± 69.4 ^a^	143.9 ± 69.0 ^a^	<0.001
Protein energy (%)	15.3 ± 6.7	15.7 ± 7.1 ^a^	15.8 ± 6.5	16.8 ± 5.5	20.7 ± 7.5 ^a^	21.0 ± 8.9 ^a^	0.001
Fat (g)	60.8 ± 26.8	53.9 ± 22.6 ^a^	49.5 ± 19.8 ^ab^	55.0 ± 26.0	54.2 ± 26.0	51.9 ± 25.8 ^a^	0.82
Fat energy (%)	21.9 ± 8.5	21.8 ± 7.8	21.9 ± 8.0	18.6 ± 6.2	17.6 ± 6.3 ^a^	16.7 ± 6.1 ^ab^	0.002
Life style							
Physical act (freq)	3.2 ± 1.4	3.3 ± 1.3	3.2 ± 1.3	3.1 ± 1.4	2.9 ± 1.3	3.1 ± 1.4	0.22

Data presented as mean ± SD; % weight change presented as median (range); superscript “^a^” denotes significance compared to baseline within group; superscript “^b^” denotes significance compared to 3 months within group; last column *p*-value denotes between-group interactions which shows the use of subsequent testing for main interaction effects; all significant *p*-values highlighted in bold; significant at *p* < 0.05. BMI, body mass index; BP, blood pressure; Chol, cholesterol; CHO, Carbohydrate; freq, frequency.

## Supplementary Materials

The following are available online at https://www.mdpi.com/2072-6643/11/5/1113/s1, Table S1: Demographic and obstetric/gynaecological characteristics of all participants, along with the house hold income, current job and the educational level of the participants.
