# Supplementary material for: Identification of Education Models to Improve Health Outcomes in Arab Women with Pre-Diabetes
Source: Nutrients. 2019 May 18;11(5):1113. doi: 10.3390/nu11051113 (PMC6566809; doi:10.3390/nu11051113)
Supplement: Supplementary file 1 [file nutrients-11-01113-s001.pdf]

Supplementary Material:

Table S1. Demographic and obstetric/gynaecological characteristics of all participants, along with the house hold income, current job and the educational level of the participants.

Parameters	Mean ± SD	Intervention	Control	*p*-Value
N	123	74	49	
Age (years)	40.6 ± 9.8	40.6±9.8	40.6±12.7	0.98
Menarche age (years)	12.7 ± 1.8	12.8±1.6	12.7±2.0	0.82
Menopausal age (years)	46.4 ± 1.7	46.1±1.6	46.9±1.8	0.12
Location (%)				
Urban	72 (58.5)	44 (59.5)	28 (57.1)	0.85
Rural	51 (41.5)	30 (40.5)	21 (42.9)	
Marital Status				
Married	86 (69.9)	53 (71.6)	33 (67.3)	0.46
Single	32 (26.0)	17 (23.0)	15 (30.6)	
Widow	5 (4.1)	4 (5.4)	1 (2.0)	
Family history of DM				
Yes	67 (54.5)	39 (52.7)	28 (57.1)	0.71
No	56 (45.5)	35 (47.3)	21 (42.9)	
Relative Degree				
First degree	67 (54.5)	39 (52.7)	28 (57.1)	0.35
Second degree	56 (45.5)	35 (47.3)	21 (42.9)	
GDM History				
Yes	12 (30.8)	12 (37.5)	0 (0)	0.08
No	27 (69.2)	20 (62.5)	7 (100)	
Income/month				
<5000 SR	23 (18.7)	10 (13.5)	13 (26.5)	0.004
(5000–10,000 SR)	36 (29.3)	16 (21.6)	20 (40.8)	
(10,000–20,000 SR)	44 (35.8)	31 (41.9)	13 (26.5)	
>20,000 SR	20 (16.3)	17 (23.0)	3 (6.1)	
Current job				
Government	64 (52.0)	37 (50.0)	27 (55.1)	0.73
Private	39 (31.7)	23 (31.1)	16 (32.7)	
Retired	12 (9.8)	9 (12.2)	3 (6.1)	
No work	8 (6.5)	5 (6.8)	3 (6.1)	
Education level				
Illiterate	7 (5.7)	2 (2.8)	5 (10.2)	0.12
Read and write	7 (5.7)	5 (6.8)	2 (4.1)	
Primary school	7 (5.7)	4 (5.4)	3 (6.1)	
Elementary school	9 (7.3)	5 (6.8)	4 (8.2)	
Secondary school	21 (17.1)	8 (10.8)	13 (26.5)	
College school	24 (19.5)	17 (23.0)	7 (14.3)	
University graduate	24 (19.5)	17 (23.0)	7 (14.3)	
Post-graduate studies	24 (19.5)	16 (21.6)	8 (16.3)	
Number of children				
None	14 (11.4)	11 (14.9)	3 (6.1)	0.03
1–3	37 (30.1)	25 (33.8)	12 (24.5)	
4–6	43 (35.0)	27 (36.5)	16 (32.7)	
≥7	29 (23.6)	11 (14.9)	18 (36.7)	

Data presented as mean ± SD for continuous variables and frequency (%) for categorical variables. Data presented in brackets represents percentages.
